# Repressive Control of Keratinocyte Cytoplasmic Inflammatory Signaling

**DOI:** 10.3390/ijms241511943

**Published:** 2023-07-26

**Authors:** Liam E. Carman, Michael L. Samulevich, Brian J. Aneskievich

**Affiliations:** 1 Graduate Program in Pharmacology & Toxicology, University of Connecticut, Storrs, CT 06269-3092, USA; liam.carman@uconn.edu (L.E.C.); michael.samulevich@uconn.edu (M.L.S.); 2 Department of Pharmaceutical Sciences, School of Pharmacy, University of Connecticut, Storrs, CT 06269-3092, USA

**Keywords:** A20, inflammation, keratinocyte, repression, TNIP1, ubiquitin

## Abstract

The overactivity of keratinocyte cytoplasmic signaling contributes to several cutaneous inflammatory and immune pathologies. An important emerging complement to proteins responsible for this overactivity is signal repression brought about by several proteins and protein complexes with the native role of limiting inflammation. The signaling repression by these proteins distinguishes them from transmembrane receptors, kinases, and inflammasomes, which drive inflammation. For these proteins, defects or deficiencies, whether naturally arising or in experimentally engineered skin inflammation models, have clearly linked them to maintaining keratinocytes in a non-activated state or returning cells to a post-inflamed state after a signaling event. Thus, together, these proteins help to resolve acute inflammatory responses or limit the development of chronic cutaneous inflammatory disease. We present here an integrated set of demonstrated or potentially inflammation-repressive proteins or protein complexes (linear ubiquitin chain assembly complex [LUBAC], cylindromatosis lysine 63 deubiquitinase [CYLD], tumor necrosis factor alpha-induced protein 3-interacting protein 1 [TNIP1], A20, and OTULIN) for a comprehensive view of cytoplasmic signaling highlighting protein players repressing inflammation as the needed counterpoints to signal activators and amplifiers. Ebb and flow of players on both sides of this inflammation equation would be of physiological advantage to allow acute response to damage or pathogens and yet guard against chronic inflammatory disease. Further investigation of the players responsible for repressing cytoplasmic signaling would be foundational to developing new chemical-entity pharmacologics to stabilize or enhance their function when clinical intervention is needed to restore balance.

## 1. Introduction

The epidermal compartment of skin is widely recognized as a constantly renewing physical barrier derived from specialized intracellular and intercellular proteins expressed by keratinocytes, the major cell type of the epidermis [1,2,3]. There is now also increasing appreciation that epidermal keratinocytes play a significant functional role in recognizing, processing, and transmitting signals from external, environmentally derived events and factors (e.g., physical damage, cutaneous pathogens, and toxins) and locally generated secretagogues (e.g., cell debris, cytokines, and chemokines) [4,5]. This aspect of keratinocytes to be both respondents to and producers of such signals sets them directly in line to dramatically influence immune and inflammatory reactions within the epidermis to neighboring keratinocytes and resident immune cells as well as underlying connective and vascular tissue.

Addressing progression of signaling, there are several excellent recent reviews covering keratinocyte roles in promoting tissue inflammation in general and specific cutaneous diseases and wound healing [4,6,7,8]. Here we sought to complement these reports by assessing intracellular components and pathways of epidermal keratinocytes that function as inflammation or immune-response repressors. Within this theme of limiting signaling, we briefly review the nature of some typically keratinocyte-encountered initiating signals, receptors that transduce them, intracellular components involved in their advancement, and, especially, proteins and complexes for their repression. In particular, we emphasize consequences to the keratinocytes and epidermis when these repressor proteins or pathways are missing or defective through experimentally introduced or naturally occurring genetic alterations. Additionally, given the repeat appearance of ubiquitin-binding for several of these proteins and/or their partners, we highlight their further recognition as writers, readers, and/or erasers within the ubiquitin code [9,10,11,12,13]. Importantly, with this, is the caveat that as research on individual proteins continues across analysis in vitro (e.g., isolated recombinant proteins) and in vivo (e.g., knockdown and genetic knockout), absolute category distinctions for some proteins may blur. An example of this is A20, now recognized for its zinc-finger (ZnF) 4 E3 ubiquitin ligase writer activity, ZnF7 interaction with methionine (M) 1-linked polyubiquitin-reader function, and N-terminal OTU domain with deubiquitinating eraser capability (see [14] for review).

## 2. Keratinocyte Inflammatory Signal Progression

### 2.1. Keratinocyte Post-Receptor Inflammatory Signaling

Inflammatory signals come in myriad forms from pro-inflammatory cytokines to molecular patterns. Among these are the pro-inflammatory cytokines interleukin-17 (IL-17) and tumor necrosis factor α (TNFα) which will be emphasized below. Such cytokines bind to transmembrane receptors that are highly specific to the particular cytokine, e.g., TNFα to tumor necrosis factor receptor 1 and 2 (TNFR1/2). Alternatively, pathogen-associated molecular patterns (PAMPs) and damage-associated molecular patterns (DAMPs) are intercepted by a variety of pattern recognition receptors (PRRs) to promote inflammation. Of the various PRRs, NOD-like receptors (NLRs) and toll-like receptors (TLRs) will be emphasized in this analysis.

**Figure 1:**
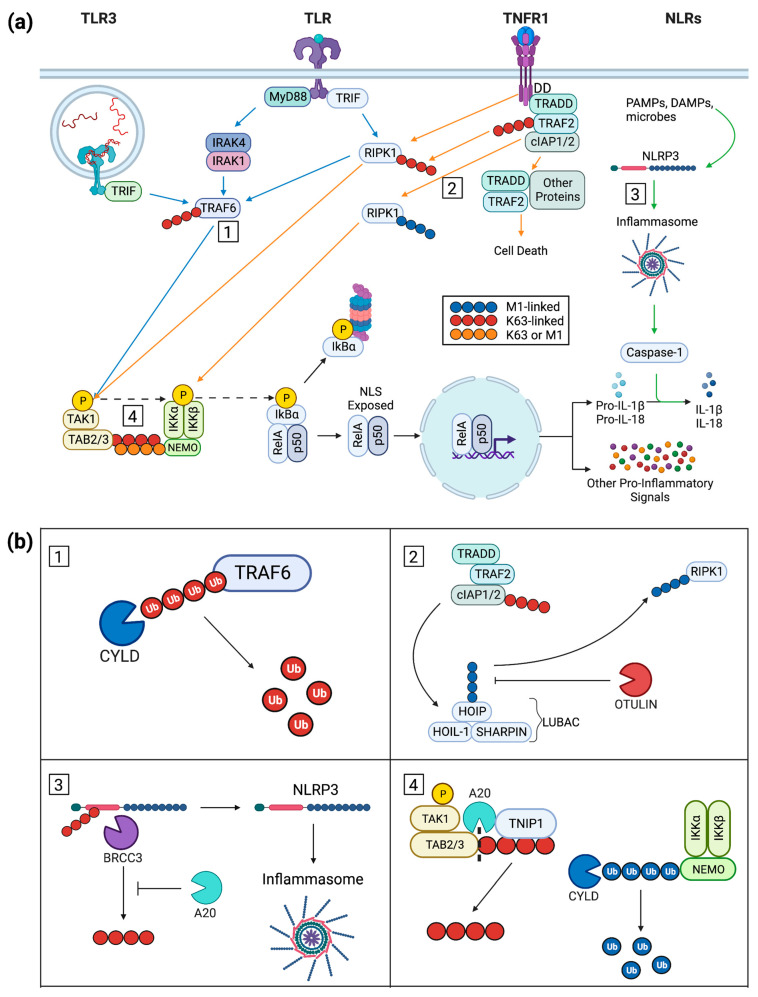
(**a**) Inflammatory signaling initiated via TLRs, NLRs, and TNFR with downstream canonical activation of NF-κB. TLR pathways are indicated by blue arrows while NLR and TNFR pathways are indicated by green and orange arrows, respectively. TLR3 and surface TLR pathways intersect with TRAF6 recruitment while the surface TLR and TNFR1 pathways intersect with K63-polyubiquitin-modified RIPK1. Also, TRAF6 and K63-polyubiquitin-modified RIPK1 overlap in their phosphorylation of TAK1. Boxed numbers 1–4 denote points of function for proteins, see (**b**), covered in this review. (**b**) Functional effects of inflammatory signaling repressors on pathways in (**a**). (**b1**) The DUB CYLD removes K63-linked polyubiquitin from TRAF6 via internal chain cleavages and ultimately reverts it to ubiquitin monomers. This prevents TRAF6 from recruiting TAK1/TAB2/3 and the progression of NF-κB signaling. (**b2**) LUBAC, composed of HOIP, HOIL-1, and SHARPIN, interacts with complex I (TRADD, TRAF2, and cIAP1/2) to form an M1-linked polyubiquitin chain used to modify RIPK1. The DUB OTULIN inhibits ubiquitination of RIPK1 by hydrolyzing M1-linked polyubiquitin to both remove it from LUBAC and process it into monomeric ubiquitin. (**b3**) A20 represses the function of the DUB BRCC3 which would otherwise remove the K63-linked polyubiquitin chain from NLRP3 leading to its activation and promotion of inflammasome formation. (**b4**) TNIP1 exhibits ubiquitin-reader activity by recognizing a K63-linked polyubiquitin chain on TAB2/3 allowing its protein partner A20 access to the chain for hydrolytic removal. CYLD can prevent the TAK1/TAB2/3 dimer from interacting with the IKK complex through its cleavage of M1-linked polyubiquitin present on NEMO. The non-canonical NF-κB signaling pathway was not included in this diagram as it is mostly outside the scope of this review. The non-canonical pathway and NF-κB in inflammation in general have been previously reviewed [35,36]. Citation: Created with BioRender.com.

Specific cytokine receptors such as TNFR1 can lead to activation of NF-κB. The TNFR1 activation pathway is immediately distinct from PRR signaling as TNF-binding triggers a trimerization of TNFR1 (Figure 1a) [15]. The cytoplasmic tail of the trimeric ligand-receptor entity serves as a central dock for TNFR1-associated signaling complex (complex I) assembly, a feat facilitated by multiple protein–protein interactions and modifications such as ubiquitination and phosphorylation (Figure 1a) [16,17]. The first complex member recruited is TNFR1-associated death domain (TRADD), a protein recruited to the death domain (DD) present on the receptor’s cytoplasmic tail (Figure 1a). TRADD serves as a base for continuation of complex I assembly with TNF receptor-associated factor 2 (TRAF2) recruited along with cellular inhibitor of apoptosis protein 1 and 2 (cIAP1/2) and receptor-interacting serine-threonine-protein kinase 1 (RIPK1). TRAF2 and cIAP1/2 have lysine (K) 63-specific E3 ubiquitin ligase activity (Figure 1a) [18,19,20], allowing them to contribute to complex I modification via addition of polyubiquitin chains to RIPK1. The now-modified RIPK1 utilizes its K63 polyubiquitin chain to bring TGF-β-activated kinase 1 (TAK1) into phosphorylating proximity via another polyubiquitin chain on TAK1 partner TAK1-binding proteins 2 and 3 (TAB2/3) (Figure 1a) [21,22]. The polyubiquitin chain of TAB2/3 is then utilized to bring the three-member inhibitor of κB kinase (IKK) complex into the assembly and allow TAK1 to phosphorylate IKKα/IKKβ [23]. Alternatively, the linear ubiquitin chain assembly complex (LUBAC) can interact with complex I to facilitate the addition of an M1-linked polyubiquitin chain to RIPK1, allowing the modified kinase to directly activate the IKK complex (Figure 1(b2)) [24].

Cellular surveillance of inflammatory signals by TNFR is complemented by NLRs and TLRs. NLRs are cytoplasmically located PRRs that detect endogenous DAMPs as well as intracellular pathogens. When prototypical NLR family pyrin domain-containing proteins (NLRPs), caspase-activating members of the NLR family, are stimulated, they form oligomeric complexes termed inflammasomes (Figure 1a) [25]. The NLRP inflammasome activates caspase-1 (Figure 1a) which facilitates cleavage of NF-κB transcription products such as pro-IL-1β and pro-IL-18 into active IL-1β and IL-18, respectively (Figure 1a) [22,26]. TLRs are PRRs that can also lead to NF-κB activation. These receptors are located both on the cell surface (TLRs 1, 2, 4, 5, and 6) and on endosomal membranes (TLRs 3, 7, 8, and 9) to recognize extracellular or phagocytosed molecular patterns, respectively [27]. A distinguishing feature of TLRs is the presence of a toll IL-1R (TIR) domain on their cytoplasmic tail. Activated TLRs form dimers allowing the TIR to recruit one of a selection of adaptor proteins such as myeloid differentiation primary response 88 (MyD88), TIR domain-containing adaptor-inducing interferon-β (TRIF), or translocating chain-associating membrane protein (TRAM) (Figure 1a) [28]. With the exception of TLR3, the prototypical TIR adapting protein is MyD88 (Figure 1a) [22]. The resulting cytoplasmic signaling cascade phosphorylates interleukin-1 receptor-associated kinase 4 (IRAK4) and 1 (IRAK1) which, complexed with TRAF6, are brought into a phosphorylating proximity with TAK1 via its partner protein TAB2/3 (Figure 1a) [21]. At this point, the MyD88-facilitated TLR pathway has intersected with the TNFR1 pathway above.

Although TLR3 is mostly localized to endosomal membranes, it can also be found at the plasma membrane of some cells [29]. As with the other TLRs, TLR3 straddles DAMP and PAMP recognition. For instance, dsRNA as a TLR3 ligand may be released from locally damaged cells or infecting viral pathogens (Figure 1a) [30]. TLR3 ligand engagement and subsequent receptor dimerization [31,32] initiates assembly of the TLR3 signaling complex (TLR3-SC) at the cytoplasmic tail of the receptor (Figure 1a). Further multimerization likely occurs among the activated dimers, enhancing overall signal transfer [32,33]. As is the case with all TLRs, a cytoplasmic TIR domain recruits TIR-containing adaptor proteins. Contrary to the example above, however, TLR3 canonically uses the adaptor protein TRIF (Figure 1a). The signaling pathway progresses as TRIF is able to recruit RIPK1, TRAF6, and cIAP1/2 with their K63-specific E3 ubiquitin ligase activity, modifying RIPK1 with a K63-linked polyubiquitin chain. In similar fashion to the TNFR1 signaling pathway, TAK1 is phosphorylated and proceeds to phosphorylate the IKK complex.

### 2.2. An Overview of NF-κB Signaling

Cytokine receptors and PRRs both converge in their interaction with NF-κB signaling. In the cases of cytokine receptors and TLRs, NF-κB signaling convergence results in IKK complex phosphorylation. However, for NLRs, the NF-κB interaction occurs primarily with the activation of NF-κB-induced cytokines.

The IKK complex is a trimeric structure composed of enzymatically active IKKα/IKKβ and their regulatory partner IKKγ (NF-κB essential modulator, NEMO). When TNFR1 signaling and TLR signaling converge at TAK1 phosphorylation, the stage is set for TAK1’s partner protein TAB2/3 to use its K63-linked polyubiquitin chain as an anchor to NEMO’s polyubiquitin chain. The polyubiquitin scaffold formation allows TAK1 to phosphorylate IKKα/IKKβ which will advance the signal to the NF-κB complex (Figure 1a).

The NF-κB family of proteins is comprised of five members in mammals; these are RelA (alias p65), RelB, c-Rel, p105/p50 (alias NF-κB1), and p100/52 (alias NF-κB2) [34]. These nuclear factors may dimerize in as many as 15 combinations to provide a variety of effects on transcription, although the RelA/p50 dimer is most abundant [34]. NF-κB dimers are regulated by members of the IκB family of proteins such as the prototypical IκBα. IκB proteins serve to restrict NF-κB dimer translocation by covering the nuclear localization signal (NLS) present on NF-κB dimers.

In the canonical NF-κB pathway, an NF-κB dimer of RelA/p50 is restricted from nuclear translocation by IκBα. The phosphorylated IKK complex promotes the NF-κB signaling pathway via phosphorylation of IκBα which is subsequently modified with a branching K48-linked polyubiquitin chain, a modification which serves as a proteasomal degradation signal (Figure 1a). As IκBα is removed, the NLS present on the RelA/p50 dimer is exposed, causing the dimer to translocate into the nucleus (Figure 1a). Here, the dimer directly interacts with DNA as an activating transcription factor within the promoter of pro-inflammatory genes, e.g., those encoding pro-IL-1β and pro-IL-18. Their protein products are then cleaved into their active form by caspase-1 downstream of NLRP (Figure 1a) [37]. Consequences of NF-κB activation in epidermal keratinocytes thus overlap with those in immune cells with production of numerous cytokines and chemokines from TNFR and TLR occupation. While here we focus on inflammation (Table 1), NF-κB signaling is also tied to the balance of epidermal keratinocyte proliferation, cell-specific gene expression, and differentiation [38,39].

### 2.3. Ubiquitin in Cytoplasmic Signaling

One of the coordinating events that is common to several of the above-mentioned receptors mediating inflammatory signals is post-translational modification by ubiquitination of the receptors themselves and downstream signal carriers. Ubiquitin is a ubiquitously expressed, 76-amino-acid modifier protein that is most often linked to form polyubiquitin chains. The formation of these chains typically requires three classes of enzymes: an E1 enzyme which activates ubiquitin, an E2 ubiquitin-conjugating enzyme, and the aforementioned E3 ubiquitin ligases [40]. There are eight different types of linkages that can connect the subunits of a polyubiquitin chain. Seven of these are internal lysine linkages (K6, K11, K27, K29, K33, K48, and K63) while one is the N-terminal methionine (M1) [40]. Of these, K48 and K63 are the most well-studied while M1 linkages have more recently been established as having physiological relevance [41].

The function of polyubiquitin chains has classically been understood to be a signal for proteasomal degradation; however, modifications have been shown to serve different purposes including scaffolding and cellular localization. K48-linked polyubiquitin chains serve as the universal proteasomal degradation signal when covalently bound to a lysine residue on a target protein. K63-linked and M1-linked polyubiquitin chains, alternatively, serve as scaffolds, mainly interacting with single proteins or protein complexes through hydrophobic interactions with specialized ubiquitin-recognizing domains such as are found in A20 and TNIP1. However, these linkages can be differentiated in part by the signaling they partake in; K63 polyubiquitin chains commonly act as scaffolds in signaling cascades while M1 chains are specifically associated with immune and inflammatory signaling [40].

The cellular ubiquitin machinery and associated proteins can be categorized into three broad categories: readers, writers, and erasers. Readers are proteins that recognize polyubiquitin chains via ubiquitin-interacting motifs, which allows partner proteins access to the chain as in the case of reader TNIP1 and partner A20 [40,42]. Writers are ubiquitin ligases which conjugate polyubiquitin chains to a substrate, be it to a target protein for ubiquitination or ubiquitin monomers, to form a polyubiquitin chain [43]. One such writer is the LUBAC which is the only identified ligase complex for M1-linked (“linear”) polyubiquitin chains [11,44]. Lastly, erasers are deubiquitinase enzymes that cleave polyubiquitin chains by hydrolyzing the chain internally (e.g., CYLD and OTULIN) or removing an intact polyubiquitin chain from a target protein (e.g., A20) (Figure 1b) [13,45,46]. It should be noted that these categories are not mutually exclusive, with some proteins (e.g., A20) acting as a reader, writer, and eraser [14]. 

## 3. Inflammation-Repressing Proteins

### 3.1. LUBAC Modulates Signaling Downstream of TNFR and TLR3 

The linear ubiquitin chain assembly complex (LUBAC) enters into post-TNFR signaling via interaction with polyubiquitin chains on RIPK1 and cIAP1/2 [47,48]. The three subunits of LUBAC (SHARPIN, HOIL-1, and HOIP) together provide scaffolding and E3 ligase activity for polyubiquitin assembly in a linear, head-to-tail manner, where the C-terminal glycine of the ubiquitin to be added is attached to the N-terminal methionine of the acceptor ubiquitin (Figure 1(b2)) [13,40,49]. Importantly, for balance of ubiquitin consequences, HOIP can also recruit OTULIN and CYLD deubiquitinases [50]. M1-ubiquitination of complex I members include RIPK1 and the receptor itself [51,52]. IKKα/β-NEMO is then recruited. Following TLR3 ligand-binding, K63 ubiquitination of the TRIF/RIPK1 assembly recruits LUBAC and its M1-linking activity. Adding to variation on the theme, LUBAC can also add M1-linked ubiquitin to existing K63 modifications [52]. The diversity of these linkages may allow for recruitment of activating (e.g., TAK) and repressive (e.g., A20 and TNIP1) protein partners [53,54]. 

### 3.2. SHARPIN 

#### 3.2.1. Mouse Mutations and Human Disease Analogues

Spontaneous mutations giving rise to dermatitis and internal organ inflammation in two laboratory mouse strains helped lead to the identification of SHANK-associated RH domain-interacting protein (SHARPIN) (Table 2). The mouse strains share a phenotype of chronic proliferative dermatitis (cpdm) from two different mutations of the gene (*Sharpin^cpdm^* and *Sharpin^cpdm-Dem^*) resulting in disruption of the protein coding sequence and premature translation termination [55,56]. In addition to a cutaneous phenotype of epidermal hyperplasia and defects in keratinization, there is wide-spread inflammatory pathology including significant increase in spleen size and liver infiltration by neutrophils and macrophages. The keratinocyte changes as well as the accompanying connective tissue infiltration with immune cells extend to other stratified squamous epithelia such as the oral cavity [57]. TNFR1 activity is directly required for the skin inflammation in cpdm mice as SHARPIN-null animals that were also null for *Tnfrsf1a* did not develop cutaneous inflammation [58,59].

There is decreased epidermal keratinocyte SHARPIN staining in biopsies from atopic dermatitis (AD) patients compared to control samples [60]. Interestingly, there are some additional parallels between cutaneous phenotypes in SHARPIN-defective mice and clinical presentation of AD with shared disrupted keratinocyte differentiation, cytokine production, e.g., IL-18, and immune-cell infiltration [57,61,62]. However, distinct from patient AD, the mouse dermatitis model appears to develop without reliance on eosinophils or signaling via receptors for interleukins 4 and 13 [63,64]. The degree to which single mutations in mouse models fully recapitulate the lesional presentation from interplay of patient genetic and environmental factors in AD will require further refinement. Although noted for control over NF-κB regarding inflammatory signaling, there is no OMIM entry for SHARPIN linking it to a human disease.

#### 3.2.2. Recombinant Models and Possible Translational Applications 

Skin inflammation is a dynamic interplay within an integrated organ of differentiated and basal replicating keratinocytes in the upper epidermal compartment with fibroblasts and microvascular endothelial cells in the lower dermal compartment plus resident and infiltrating immune cells within both. Studying events intrinsic to keratinocytes, and, in turn, what effect modified keratinocytes might have in their tissue environment, has been greatly aided by the availability of transgenic mouse lines with Cre recombinase under control of largely cell-specific gene promoters such as the basally expressed keratin 14 (K14) [65].

Utilizing a K14-Cre approach, SHARPIN has been selectively deleted from mouse keratinocytes [57]. Overall, the cutaneous presentation seen in the two spontaneous loss-of-function mouse strains was recapitulated. There is a dermatitis phenotype involving epidermal hyperplasia across the spinous layer (acanthosis) and the cornified layer where some cells exhibited delayed or defective maturation marked by nuclei retention (parakeratosis). Apoptotic keratinocytes histologically marked by nuclear condensation and blebbing are present. There is epidermal and dermal infiltration with inflammatory cells (eosinophils and neutrophils). However, SHARPIN loss from the K14-Cre-mediated recombinant event is less dramatic in other stratified epithelia such as the esophagus or forestomach where SHARPIN is natively expressed in wildtype mice and where the K14 promoter is expected to be active. In contrast, deletion of SHARPIN dependent on the ubiquitously active CMV-Cre cassette does mirror the histologic lesions in these tissues seen in the spontaneous loss-of-function strain *Sharpin^cpdm^* where an early stop codon results from a mutational reading frame shift. We venture that these variances may stem from the local tissue microenvironment and/or innate differences in keratinocytes at these sites for expressing the promoter driving Cre. Additionally, skin keratinocytes may be more inherently sensitive to SHARPIN loss, whereas those in other stratified epithelia require the intrinsic loss of SHARPIN and intercellular events or signals generated by neighboring SHARPIN-null cells (fibroblast, endothelial cells, etc.). This is supported by earlier work of transplanting *cpdm* skin to *nude* mice where the dermatitis phenotype of the donor skin was maintained [66].

Demonstrating the breadth of SHARPIN involvement in addition to post-TNFR1 signaling, *Sharpin^cpdm^* mice null for TLR3 had a very much reduced cutaneous inflammatory phenotype [67]. Walczak and colleagues [67] posited that TLR3 and its ligands, e.g., self-nucleic acids acting as endogenous danger signals and released as part of TNFR-mediated cell death, may be contributing to the inflammatory response. Epidermal presence of the hyperproliferative marker keratin 6 and dermal infiltrates of immune CD45 cells seen in the *Sharpin^cpdm^* mice was almost completely negated when those mice are also null for TLR3. Epidermal thickness was greatly reduced but not completely normalized. Keratinocyte cell death, likely to have been providing inflammatory triggers, was also reduced. These findings are particularly revelatory for the importance of additive and possibly synergistic factors that may contribute to overall cutaneous phenotype in the *Sharpin^cpdm^* and, possibly, keratinocyte-targeted models. Increased sensitivity to TLR3 activation also occurred with our TNIP1 knockdown [68] in human keratinocytes despite these two proteins acting in very different steps of inflammatory signaling.

SHARPIN levels have also been manipulated in cultured keratinocytes. SHARPIN knockdown [60] in the human non-tumorigenic HaCaT keratinocyte line resulted in an ~2-fold increase in IL-33 expression which may have been dependent on increases in activated (phosphorylated) JAK/STAT signaling components [69]. This SHARPIN deficiency result parallels the IL-33 increase reported for AD [70,71]. In the epidermis, this keratinocyte-derived cytokine increases subsequent to barrier disruption, possibly more-so from increased processing of constitutively produced inactive precursor. In turn, it can activate resident immune cells [72,73] and contribute to the dermatitis presentation. 

Although several steps downstream of a possible SHARPIN deficit-initiated pathology, initial studies with an anti-IL-33 monoclonal antibody (etokimab) showed promising results for reduction in skin inflammation associated with AD ([74]; reviewed in [75]). However, an overview [76] of that monoclonal antibody directed against IL-33 itself and another (astegolimab) directed against its ST2 receptor [77] reiterated that while this ligand-receptor signaling is associated with AD, later phase trials of each have not seen improved clinical outcomes [78,79]. It may be that the disease was established beyond a point when clinical intervention against IL-33 signaling alone could bring about sufficient reduction of inflammatory signaling arising from multiple, non-IL-33 players in established lesions. These results highlight that therapeutic targeting of any possible AD contributing factor, such as SHARPIN deficiency, may need to be implemented based on biomarkers detected earlier than overt chronic pathology.

### 3.3. HOIL-1 and HOIP

#### 3.3.1. Human Pathologies and Protein Function

SHARPIN has been intensely investigated as a non-catalytic subunit of the LUBAC. Its partner proteins in LUBAC are heme-oxidized IRP2 ubiquitin ligase 1 (HOIL-1) and HOIL-1-interacting protein (HOIP, also known as ring-finger protein 31, RNF31), an E3 ubiquitin ligase. Despite the enzymatic activity of both, the production of M1-linkages is attributed to HOIP [80,81,82]. This helps to understand that there is not complete mimicry in phenotype upon deletion of individual LUBAC components.

In humans, mutations in HOIL-1 can result in a weakening myopathy, severe immunodeficiency, and a hyper-inflammation (OMIM, #615895). Acute febrile neutrophilic dermatosis (fever with accumulation of neutrophils in the skin) has been reported to accompany a patient’s HOIL-1 mutation [83]. Other HOIL-1 loss-of-function mutations have been described [84] with systemic autoinflammation but with no remarks on specific cutaneous effects.

A point mutation in HOIP [85] appears to be causative for a patient’s systemic autoinflammation and combined immunodeficiency. Although numerous organ systems were affected, e.g., oral ulcers, splenomegaly, and respiratory distress, specific cutaneous effects other than persistent warts were not reported. 

#### 3.3.2. Recombinant Models

In contrast to the inflammatory phenotype from the various SHARPIN disruptions in mice above, an initial study of HOIL-1-targeted mice reported complete embryonic development, no macroscopic effects in weanlings, but some cellular level signaling defects including increased TNF-induced JNK activity [86] although this may have been cell-type dependent [87]. Additionally, for whole-animal-null studies, the degree of effect may be due to the initial strategy of targeting exons 7 and 8 [86] possibly producing a C-terminally truncated protein providing some HOIL functionality if the N-terminal and central ubiquitin-binding domain were still available [88]. HOIL-1-deficient mice generated by targeting exons 1 and 2 [88] died at approximately embryonic day 10.5. A TNFR1-null background rescued the early embryonic lethality, but nevertheless cardiac defects led to death at approximately embryonic day 16.5. Whether observed or not at this time-point, cutaneous effects were not reported.

Mice homozygous for keratinocyte-specific deletion of HOIL-1 via activity of the K14-Cre recombinase system completed development, appearing normal at birth and up to about postnatal day 2 [24]. However, epidermal defects manifested and became lethal by postnatal day 6. There was epidermal thickening, incomplete differentiation, apoptotic cell death, and immune-cell infiltration (CD45+) to both the epidermal and dermal compartments. Epidermis-deleted HOIL-1 mice that were also null for TNFR1 had delayed development of the cutaneous defects.

Mice null for HOIP are embryonic lethal due to TNFR1-mediated excess signaling leading to cell death in developing endothelia [89] although HOIP is ubiquitously expressed. Using a K14-Cre-mediated approach, epidermis-specific knockout of HOIP was achieved [24,90] allowing full embryonic development but with 4–6-day postnatal lethality due to severe skin inflammation similar to the HOIL-1 epidermal targeted deletion above. There was epidermal hyperplasia concurrent with reduction of suprabasal differentiation markers keratin 10 and loricrin by postnatal day 5. Gene expression analysis via RNA-seq from total skin tissue RNA (thus a likely mixture of keratinocyte and non-keratinocyte cells) showed enrichment for numerous interleukins, including IL-33, among increased levels of other inflammatory response and mediator transcripts downstream of TNFR1 signaling. Akin to the rescue of the HOIP-germline-null mice by a TNFR1^-/-^ background [89], skin inflammation did not develop with the keratinocyte-targeted HOIP-deletion-absent TNFR. Normal epidermal differentiation gauged by suprabasal markers was present at postnatal day 21 [90] although this may have been age-dependent. At postnatal day 70, epidermal hyperplasia was apparent judged by extensive hyperkeratosis and keratin 6 expression [24]. 

### 3.4. OTULIN

#### 3.4.1. Human Pathologies and Protein Function

OTULIN (ovarian tumor deubiquitinase with linear linkage specificity) mutation in humans causes severe systemic autoinflammatory disease including skin rash and cutaneous inflammatory cell infiltration [91,92]. Homozygous mutation in the OTULIN gene is causative for AIPDS-autoinflammation, panniculitis, and dermatosis syndrome (OMIM, #617099) [93] alternatively referred to as ORAS-OTULIN-related autoinflammatory syndrome. AIPDS/ORAS is a potentially fatal OTULIN mutant, autosomal recessive autoinflammatory disease displaying sterile systemic inflammation, recurrent high fevers, and, for skin-related presentations, includes inflammation of the subcutaneous adipose (panniculitis) and erythematous rash (Table 3) [91,92,93].

**Table 2:** Summary of epidermal or keratinocyte presentation from example alterations in LUBAC genes associated with limiting or repressing cytoplasmic inflammatory signaling.

Gene ^#^	Alteration *	Keratinocyte/Epidermal or Other Presentation ^	Refs.^$^
*Sharpin* *^cpdm^*	mSP: 1 base pair deletion in exon 1 disrupts reading frame generating early stop codon	Chronic proliferative dermatitis; immune-cell accumulation in skin with epidermal hyperplasia and keratinization defects	[55,56,57]
*Sharpin* *^cpdm-Dem^*	mSP: 14 base pair deletion in exon 1 disrupts reading frame generating early stop codon	Dermatitis, epidermal hyperplasia, and keratinization defects similar to cpdm	[56]
*Sharpin*	mEKO: K14-Cre-mediated deletion	Acanthosis and parakeratosis; apoptotic keratinocytes and dermatitis similar to cpdm	[57,61]
*SHARPIN*	cKCKD	SHARPIN-deficient HaCaT keratinocytes; increased STAT-dependent transcription and increased IL-33	[60,69]
*HOIL-1*	HG: mutations resulting in truncation of or frameshift within coding region	Febrile neutrophilic dermatosis; generalized immunodeficiency and hyperinflammation; polyglucosan body myopathy-1	[83,84]
*HOIL-1*	mEKO: K14-Cre-mediated deletion	Postnatal epidermal hyperplasia, defective differentiation, immune-cell infiltration to both epidermal and dermal compartments ~2 d postnatal, lethal ~6 d postnatal	[24]
*HOIP*	HG: base pair transition with amino-acid substitution	Nucleotide sequence alteration of undetermined significance, patient displayed autoinflammation and immunodeficiency	[85]
*HOIP*	mEKO: K14-Cre-mediated deletion	Postnatal skin inflammation, epidermal hyperplasia, reduced differentiation, increased expression of multiple interleukins, lethal 4–6 d postnatal	[24,90]

^#^ Mouse genes (all italics, first letter capitalized); human genes (all italics, all capitals). *Alteration. HG: human genome variation; mSP, mouse spontaneous mutation; mEKO, mouse epidermal keratinocyte-selective knockout; cKCKD; cultured keratinocyte knockdown. Descriptions of spontaneous human or murine genes are representative and not necessarily inclusive of all reports. ^ Highlights of cutaneous or cell phenotype as in text when available from the literature. ^$^ For initial report or other description of alteration.

Heterozygous OTULIN mutations (missense, nonsense, or frameshift) are associated with IMD107-immunodeficiency-107 with susceptibility to invasive *Staphylococcus aureus* infection (OMIM, #619986) [94], although there is phenotypic heterogeneity with the mutation and an overt infectious agent may not be identified. Individuals carrying such OTULIN sequence differences were susceptible to extreme inflammation of skin and/or lungs following *S. aureus* infection [94].

OTULIN, a member of the ovarian tumor subfamily of deubiquitinases (DUBs) [95], specifically cleaves M1-linked polyubiquitin, the product of ubiquitination via the LUBAC (Figure 1(b2)). LUBAC action contributes to cell signaling via protein platform assembly rather than degradation seen from proteasome action on proteins tagged with K48 ubiquitin chains. OTULIN DUB activity not only removes ubiquitin from LUBAC substrates but also chains resulting from LUBAC auto-ubiquitination [96]. Although CYLD (cylindromatosis lysine 63 deubiquitinase) and A20 also hydrolyze M1 polyubiquitin, along with K63 polyubiquitin chains, their removal of M1-linkages does not appear wholly redundant to OTULIN’s exclusive targeting of M1 polyubiquitin [97].

#### 3.4.2. Recombinant Models

In mouse models, OTULIN germline null genotypes are embryonic lethal [98]. There is a range of inflammatory phenotypes seen with deletions targeted to different immune-cell types, from no overt inflammation when deleted from T or B cells to elevated white-blood-cell counts and increased spleen and thymus sizes when deleted from myeloid cells [91]. Examining OTULIN function in cultured keratinocytes, HaCaT cells were stably transduced with small hairpin RNA to decrease endogenous OTULIN production [99] and then exposed to TNFα. Compared to control cells, HaCaT cells depleted for OTULIN showed unexpectedly reduced M1 polyubiquitin levels on NEMO and RIPK1. This may show a role for OTULIN in facilitating LUBAC, possibly by limiting its inhibitory self-ubiquitination [99] rather than overall restricting it under basal and inflammatory conditions.

A K14-Cre model was recently used by two groups [100,101] to examine effects of OTULIN loss in murine epidermal keratinocytes. Within the first week postnatal, mice with OTULIN deleted from K14-expressing cells spontaneously developed sites of cutaneous inflammation on their backs and tails macro- and microscopically distinguishable from surrounding nonlesional skin before weaning [100,101]. Keratinocytes were hyperplastic as marked by nascent keratin 6 expression and reduction of the differentiation-associated protein, keratin 10 [100,101]. This hyperplasia favored initial wound healing but later recruitment of immune cells was interpreted as eventually slowing re-epithelialization [100,101]. While OTULIN loss was from the keratinocyte genome, surrounding tissue responded to the keratinocyte alteration with increased numbers of CD11b- and F4/80-positive immune cells, cytokines, and chemokines. A CRISPR/Cas knockin of a mutated human codon OTULIN variant mirrored these murine gene knockout results supporting translation relevance of the observed inflammatory phenotype [96].

Given demarcated back skin lesions versus the much more widespread effect to tail skin, some region-inherent keratinocyte differences may have contributed to lesion manifestation in addition to the OTULIN-null state. These studies may show that despite an epidermis-wide loss of OTULIN, some differences in microenvironment signaling factor, tissue niche physical component, or topical encounter may have added to the focal phenotype perhaps mirroring clinical focal lesion occurrence in patients. It would be a particular interest to see what loss of OTULIN conferred to keratinocytes in non-epidermal stratified epithelia, e.g., esophagus, tongue, and gingiva [102,103,104,105] where K14 expression and thus successful Cre excision would be expected to occur and if this too was focal or diffuse in distribution.

### 3.5. CYLD

#### 3.5.1. Human Pathologies and Protein Function

CYLD is a deubiquitinating enzyme named for its susceptibility association with familial cylindromatosis (OMIM, #132700). Heterozygous mutations in *CYLD* are linked to clinical presentation of multiple neoplasms of skin appendages [106,107]. Despite CYLD expression in many other tissues, the growths seem restricted to skin with hair follicles or apocrine gland epithelia [108] and are usually benign but can progress to malignancy [107]. Loss of CYLD function coincident with these overgrowths and its mutation and dysfunction in several other cancers has led to consideration of it as a tumor suppressor [109,110]. As a DUB, CYLD, like OTULIN, can remove M1-linked polyubiquitin chains (Figure 1(b4)). It can also target K63-linkages on TRAF and NEMO and has limited activity reported for K48 (Figure 1(b1)) [49,111]. CYLD removal of K63 and M1 ubiquitin restricts downstream NF-κB activation, in turn limiting pro-inflammatory gene expression.

#### 3.5.2. Recombinant Models

Complementary work in cultured keratinocytes and mouse epidermal models with wildtype and deubiquitinase-defective CYLD shows influence of the protein over keratinocyte differentiation, response to cytokine exposure, and activation of NF-κB and other transcription factors [109,112,113,114,115]. HaCaT keratinocytes stably transfected to over-express CYLD have increased levels of the early differentiation markers keratin 1 and 10 [114]. HaCaT cells expressing a catalytically deficient CYLD point mutation (C601S within the cysteine box of the deubiquitinase domain) did not show the organized cell layering seen with control cultures under organotypic conditions. Also, consistent with the mutant protein competing with the endogenous CYLD DUB, there was a significant increase in levels of ubiquitinated NEMO (IKKγ) following TNFα treatment, although subsequent inflammatory markers along that pathway were not assessed. 

A mouse model expressing wildtype CYLD from a keratin-5-promoter transgene to target expression to epidermal keratinocytes showed increased detection of the late differentiation marker filaggrin and reduced activation of NF-κB signaling in skin keratinocytes [109,116]. With the keratin-5-promoter transgene driving expression of the CYLD C601S point mutant protein, there was a constitutive increase in phosphorylation of the NF-κB p65 subunit along with increased production of inflammation markers IL-6 and TNFα. Also, transient increases in phospho-p65 from TNFα exposure took longer to resolve to pre-exposure levels. Paralleling these results, HaCaT keratinocytes expressing the C601S point-mutant protein showed perturbation of signaling regulation as indicated by phospho-p65 and TNFα levels over controls [109]. These recombinant results suggest that even if of a different cellular origin than the experimentally targeted cultured or in situ epidermal keratinocytes, epithelial cells of cylindromatosis lesions may be growth-promoted by a combination of cell-intrinsic CYLD mutation effects and local paracrine inflammatory signals from associated interfollicular keratinocytes. This is supported by the likely involvement of multiple pro-inflammatory pathways linked to CYLD [107].

Separate mouse modeling for epidermal effect of CYLD assessed a K14-Cre–mediated deletion of floxxed *Cyld* exon 9 (*Cyld ^Edelta9/delta9^*) rendering the resultant protein truncated and catalytically inactive [112]. Indicative of the protein truncation effect, skin extracts showed significantly higher levels of K63-polyubiquitination of TRAF6 and phospho-IκBα than in control mice; the downstream impact on inflammation marker expression was not reported. However, response to pro-inflammatory two-stage skin carcinogenesis induction by 7,12-dimethylbenz[a]anthracene and 12-O-tetradecanoylphorbol 13-acetate showed a preference for nodules resembling cylindromatosis cylindroma and trichoepithelioma compared to papillomas in control mice [112]. These results with the dominant negative effect of the truncated protein may be more akin to those for modeling missense, deletion, and other mutations identified in patients [117] than complete CYLD-null systems.

### 3.6. A20

#### 3.6.1. Overview and Protein Function

TNFα-induced protein 3 (TNFAIP3), commonly known as A20, is a primary repressor of NF-κB signaling. A20 is present at low levels in most cells, but with its promoter under transcriptional control by NF-κB, it can also serve as a regulator through a negative-feedback mechanism [14,118]. Well-researched since its discovery over 30 years ago [119], A20 SNPs have been connected to many inflammatory diseases such as rheumatoid arthritis [120], systemic lupus erythematosus [121], and psoriasis [122]. The only monogenic disease for A20 currently described is haploinsufficiency of A20 (HA20; OMIM, #616744). HA20 was first described in 2016 and as of 2019 [123], 24 pathogenic variants of A20 had been described with 62 reported patients. HA20 often presents similarly to Behcet’s disease though significant clinical variance was reported [123] and newer autoinflammatory pathologies resulting from HA20 have been described [124,125]. Skin rash and inflammation in several other organs and tissues, e.g., respiratory tract, gastrointestinal tract, and joints, is common [126,127].

A20 is a member of the OTU family of deubiquitinases; however, the catalytic N-terminal OTU deubiquitinase domain is dispensable for A20-controlled NF-κB activity [128]. Additionally, A20 possesses both E3 ubiquitin ligase and non-enzymatic ubiquitin-recognition abilities conferred through its ZnF4 and ZnF7 domains, respectively [129,130]. Hence, it is often referred to as a ubiquitin-editing enzyme that can also function as a scaffold (reader) for coordinating ubiquitinated proteins [131]. Despite its enzymatic abilities, this scaffolding ability through ZnF7 is most vital to its ability to restrict NF-κB signaling [132]. For A20 control downstream of TNFR, a current working model includes its binding to M1-linked polyubiquitin chains on TNFR-signaling-complex I through ZnF7 [133] facilitated by its binding partner protein TNIP1 (Figure 1(b4)) [42]. This prevents the necessary downstream machinery like the IKK complex from binding to polyubiquitin chains and becoming activated through phosphorylation, restricting NF-κB signaling [132]. It also prevents dissociation of complex I which can lead to complex II formation consequent necroptosis, apoptosis, or pyroptosis (Figure 1a) [133]. Furthermore, A20 displays its repressive function in several other pattern recognition and cytokine receptor signaling pathways [134,135,136], in addition to TNFR, such as TLR discussed below.

As in other cell types, dissecting the control by A20 over skin inflammation has required separating A20 function within specific cells such as the keratinocyte from local tissue effects facilitated by A20 in associated dermal fibroblast, vascular endothelial, and resident and infiltrating immune cells [137]. A20 was found in one study to be downregulated in the epidermis but not the dermis of patients with psoriasis or atopic dermatitis (AD) [138]. It has not come up as a susceptibility locus for AD in genome-wide association studies (GWAS) though genomic findings only account for about 30% of AD heritability [139]. This disconnect between clinical presentation and definitive linkage to a specific gene alteration does not limit the impact of the gene’s protein product when it is deficient or defective, a concept generalizable to any inflammation repressor protein. This is the case regarding the apparent importance of A20 in AD from its involvement in the IL-17 axis in which the absence of A20 sees keratinocytes exhibiting enhanced inflammatory gene expression both without and with stimulation by IL-17A [138]. Supporting these findings, A20 overexpression in normal human epidermal keratinocytes (NHEKs) was shown to repress many inflammatory genes under basal conditions and an even greater number when stimulated by IL-17A; repressed genes included those encoding chemokines, psoriasins, defensins, and interleukins [140]. Exposure of A20-overexpressing NHEKs to TNFα resulted in repression of the IL-17A gene set with the inclusion of TNFα specific genes like *ICAM1*, *CSF1/2*, and *TNF*. Also noteworthy from this study was the repression of late differentiation genes like small proline-rich proteins. The spectrum of A20 effects paints a picture of its involvement in multiple cell signaling pathways and diverse consequences [141]. Repressor proteins like A20 act as lines of defense against multifactorial diseases wherein their hypomorphic or absent expression increases susceptibility to said diseases [142].

A20 is inducibly expressed in most cell types, typically within 30–60 min of NF-κB activation [143]. lncRNA UCA1, which is downregulated in the lesional skin of psoriasis patients, induces A20 expression and thus NF-κB repression by inhibiting miR125a, a transcriptional down-regulator of A20 [144]. Similarly, miR-155 inhibition by a bacterially derived exopolysaccharide increased A20 transcription leading to decreased inflammatory signaling in the IL-17A axis [145]. Additionally, certain pathogens use natural repressors like A20 to their advantage in evading host response. *Staphylococcus epidermidis* induces expression of A20 in human keratinocytes, helping protect the bacterium from NF-κB-mediated transcription of human β-defensin 2 [146]. The challenge will be to translate such control of A20 expression to new therapeutic opportunities for limiting inflammation.

#### 3.6.2. Inflammatory Cell Death

A20 could be a potential key player in cell death as it has been shown on a few occasions to inhibit caspase-8 activity by an only partially understood mechanism [147,148]. Since caspase-8 is a molecular switch inhibiting necroptosis, this might suggest A20 could promote this form of cell death. However, the role A20 and other ubiquitin-editing enzymes have in necroptosis differs [149,150] for as yet unknown reasons [151]. Ildefonso et al. present a biochemical model of necroptosis in a mouse fibrosarcoma cell line (L929) that can explain some of these differences [151]. However, the authors point out that the relevance of this model to other cell lines depends on whether they have the same set of involved proteins as in L929s. Only a few recent studies have directly investigated the effects of A20 on cell death in keratinocytes. Two studies reported that A20 deficiency led to no increased sensitization to TNF-induced cell death in both HaCaT keratinocytes [152] and mouse models [138], though the authors of the latter point out that this does occur in other cell types such as enterocytes and mouse embryonic fibroblasts (MEFs) [153,154]. Regarding MEFs, a more recent study showed A20 is able to restrict RIPK1 mediated necroptosis when it is recruited to M1-linked polyubiquitin chains by TNIP1 [42]. Conversely, A20 deficiency in gingival keratinocytes increased apoptotic response when these cells were challenged with both TNFα and cycloheximide, a reliable model for apoptosis induction [155]. Intriguingly, NF-κB inhibition in keratinocytes has been shown to cause RIPK1 mediated necroptosis and spontaneous development of severe chronic inflammation in mice [156]. In this study, NF-κB was inhibited by performing epidermal knockout of IKK2 alone or Rel-A and c-Rel. The results suggest that targeting NF-κB activation may not be the best approach to reducing cell death as some repressors such as A20 and TNIP1 [133,157] are under the transcriptional control of NF-κB [158,159]. Targeting those repressors secondary to NF-κB activation, given their multiple levels of control, might prove to be more informative. Further deepening the intrigue of caspase-8, Kumari et al. [156] point out astutely that regulation of necroptosis within the tissue microenvironment may be different from the in vitro cell culture systems. This could suggest that there is another currently unknown pathway to necroptosis in cells that could be the missing link to understanding how A20 and its partners could both promote and restrict RIPK1-mediated cell death in a context-dependent manner. While potential therapies targeting prominent activators of both types of inflammatory cell death such RIPK1/3 or MLKL have been reported [160,161], those targeting natural repressors such as A20 (see below in Section 4) and related proteins are limited suggesting a potentially interesting avenue of investigation.

#### 3.6.3. Inflammasome Regulation

A20 has been implicated in inflammasome activation not only through NF-κB regulation but also through interaction with the BRCC3-containing BRISC complex which deubiquitinates and primes NLRP3 for inflammasome activation (Figure 1(b3)) [162]. This was found from a novel loss-of-function mutation yielding HA20 known to cause widespread chronic inflammation [123,124,125,126,127]. Interestingly, BRCC3-dependent regulation of NLRP3 has emerged as a powerful regulatory mechanism and potential therapeutic target [163]. Other research has shown A20 restricts ubiquitination of pro-interleukin-1β protein complexes (associated with TNFR signaling complex) which could then be a suppressor of the NLRP3 inflammasome [164]. Given the nature of their interaction, it is possible TNIP1 could assist A20 in executing its function which in turn could be hampered by TNIP1 deficiency. This would agree with our previous report in which TNIP1-deficient HaCaT keratinocytes show greater expression of inflammasome- and pyroptosis-associated proteins (NLRP1, caspase-1, gasdermin D, IL-1β) upon TLR3 stimulation compared to their TNIP1-sufficient counterparts [165]. Myeloid specific deletion of A20 in mice (*lysm*-Cre, *a20^flox/flox^*) also results in widespread rheumatoid inflammation in an NLRP3-dependent, TNFR-independent manner [166]. Both NLRP3 and NLRP1 are highly expressed in myeloid cell lines supporting findings of this study, yet it is known that germline NLRP1 mutations mainly manifest in skin inflammatory and cancer susceptibility [167]. It is therefore of interest what specific contribution to mouse inflammatory phenotypes A20 epidermal knockout could have compounded with epidermal knockout of NLRP1 or NLRP3.

### 3.7. TNIP1

#### 3.7.1. Overview of TNIP1

TNFα-induced protein 3-interacting protein 1 (TNIP1), known also by its aliases ABIN-1 [168], NAF1 [169], and VAN [170], is a non-enzymatic ubiquitin-binding protein perhaps most noteworthy for its role in suppressing inflammatory signaling. TNIP1 is expressed in a wide variety of tissues [171,172,173,174] and performs its role downstream of TLR, TNFR, EGF-R, and IL-17 signaling [175] helping to inhibit NF-κB, IRF3, and ERK transcription of pro-inflammatory genes [165]. TNIP1 expression is likely both constitutive and under the control of inducible transcription factors such as NF-κB itself [159,176,177,178]. *TNIP1* SNPs have been reported in several GWAS publications linking it to inflammatory diseases like psoriasis [179], systemic lupus erythematosus (SLE) [180], psoriatic arthritis [179], and systemic sclerosis [181]. Top-scoring *TNIP1* sequence variations associated with inflammatory diseases are most frequently in noncoding sequences (intronic and upstream/downstream potential expression-regulatory sequences). Two *TNIP1* risk haplotypes for SLE [182] result in lower levels of TNIP1 mRNA and protein. One (rs2233290) also confers a proline to alanine substitution in the N-terminal third of the protein where the functional consequence has yet to be determined.

#### 3.7.2. Protein Function and Interactions

TNIP1 is one of five human proteins containing an aptly named ubiquitin-binding in ABIN and NEMO domain (UBAN), alongside TNIP2 (alias ABIN-2), TNIP3 (alias ABIN-3), OPTN, and NEMO itself. Through its ABIN homology domain 1, TNIP1 recognizes M1- and K63-linked polyubiquitin chains and mechanistically helps bring its partner A20 into the proximity of these chains (Figure 1(b4)) [183,184]. This TNIP1-A20 complex can then inhibit the ubiquitin-binding of other complexes such as the TAB/TAK and IKK complex which can prevent their activation and subsequent NF-κB activation (Figure 1(b4)). This may happen in either an enzymatic manner through A20’s deubiquitinase activity or a non-enzymatic manner through competitive binding of ubiquitin [23]. With TNIP1, A20 can restrict TNFR signaling by binding to M1 polyubiquitin chains on TNFR signaling complex I as well as prevent cell death by detachment of complex I [42].

Many molecular interactions have been observed with TNIP1, such as those with protein kinases like ERK2 [169], cytoplasmic signaling and adaptor proteins like NEMO [185], or TAX1BP1 [186]. Being an intrinsically disordered protein, i.e., a protein lacking fixed tertiary structure, TNIP1’s multitude of potential partners all but ensures that the protein has other yet unknown functions [187]. NLRP10 has been shown to associate with TNIP1 [188]. NLRP10 is notably highly expressed in the epidermis, upregulated in psoriasis, and linked by GWAS to dermatitis, food allergies, and contact hypersensitivity [188]. Mirza et al. [188] show that NLRP10 can reduce cytosolic levels of TNIP1 in NHEK and HeLa cells. They are, however, keen to point out that cell-line differences may lead to opposing effects and that while NLRP10 can be implicated in all cases, the mechanism of its effects can be variable and remains to be fully elucidated. Further, they suggest that since both NEMO and TNIP1 can bind NLRP10, there may exist competition between them for that protein creating a “switch mechanism” for NLRP10-mediated inflammatory response. Our observation that TNIP1-deficient HaCaT keratinocytes express NLRP10 at much higher levels upon poly(I:C) stimulation than their WT counterpart could be interpreted to support this hypothesis [165].

More recently, TNIP1’s interaction with a number of autophagy-related proteins has sparked interest in not only its degradation by autophagy but also, a potential role as a selective-autophagy receptor itself [189,190]. Autophagy is now understood to be another potential control on inflammation through its selective degradation of pathway components as well as pathogens and damaged organelles [191]. The relationship between both processes is certainly more intricate, as repressors of inflammation can also be degraded [192]. TNIP1 directly interacts with LC3, an important autophagy protein, and localizes to autophagosomes with this interaction being enhanced by TBK1 phosphorylation on amino acid S83 [190]. It has been likened to the well-known autophagy receptor p62/SQSTM1 which itself has been shown to negatively regulate keratinocyte TLR2/6 and TLR4 inflammatory pathways in a TRAF6- and MyD88-dependent manner [193]. Both p62 and TNIP1 are now understood to contribute to autophagy through their ability to bind ubiquitin and ubiquitin-associated cargo and can likely compensate for depletion of one another in parallel. The picture is complicated further by the finding that TNIP1 can actually inhibit certain forms of autophagy like mitophagy by interacting with a key activator of the pathway, FIP200 [194]. Le Guerroué and colleagues [194], citing a report by Merline and coworkers [195] showing TNIP1 as promoter of mitophagy, note that this promotion occurs at a later stage of mitophagy. This possibly explains the apparent opposing functions of TNIP1 and suggests that the time-dependent states of autophagic response, as well as their precise roles in inflammation, have yet to be fully characterized.

#### 3.7.3. Recombinant Models

Similar to A20, overexpression of TNIP1 in NHEKs repressed many inflammatory genes, albeit a more modest set than A20 [140]. Likewise, a greater number were repressed subsequent to IL-17A stimulation, including a smaller set of chemokines, psoriasins, defensins, and interleukins [140]. The largest difference between A20 and TNIP1 in Harirchian et al. [140] was in TNFα stimulation where TNIP1 only significantly repressed four of the TNFα-induced genes. TNIP1-deficient HaCaT keratinocytes show increased expression of genes for epithelial remodeling, inflammation, and inflammasome activation upon poly(I:C) exposure [165]. As such, knockout of TNIP1 in mice greatly increases their sensitivity to topically applied imiquimod in developing psoriasis-like skin symptoms [175]. K14-Cre knockout of *Tnip1* showed similar results suggesting specific epidermal contribution of TNIP1-deficient keratinocytes to the disease phenotype in an IL-17-dependent manner. The relevance of the imiquimod-driven mouse model to human psoriasis is supported by previously cited human-case-report studies [196] and CD11c- Cre/*Tnip1^flox/flox^* dendritic-cell mouse models showing similar results [197]. From this, TRAF6 appears to be an important common denominator as an E3 ligase shared in both IL-17R and TLR-pathways, activating the TAB/TAK complex and NF-κB through K63 polyubiquitin chains [198]. Many other psoriasis-susceptibility genes including *TNFAIP3* (A20) code for proteins that interact with TRAF6 [199]. Thus, reduced TNIP1 protein levels may not be wholly responsible for all keratinocyte psoriatic symptoms [200]. Instead, diminished TNIP1 may be one of several multigenic contributions to an inflammatory phenotype.

#### 3.7.4. Endogenous and Exogenous Control of TNIP1 Protein Levels

The capacity of TNIP1 to repress inflammation is derived from a complex interplay of events and factors regulating its ultimate protein levels. These include its relatively low level of basal expression in many different cells, reduced protein levels in some inflammatory disease states, and availability of partner proteins [23,159,177,178,190,201]. While *TNIP1* gene expression is positively regulated by inflammation-associated NF-κB activation, initial response to inflammatory signals includes TNIP1 protein degradation [190,202,203]. TNIP1 breakdown may be stimuli- and cell-type-dependent based on data to-date and includes proteasome- and autophagy-dependent routes [190,202,203,204]. The mechanism of regulation appears to favor limited, early inflammatory signaling. While this benefits initial immune-cell recruitment and keratinocyte activation of wound healing-associated genes, it results in eventual signal inhibition to avoid excessive acute or chronic inflammatory responses [165]. Interestingly, in Cutibacterium acnes infection of cultured keratinocytes and organotypic skin models, TNIP1 expression is increased in a bacteria-dose-dependent manner [205]. In this experimental system, all-trans retinoic acid (ATRA), a known acne treatment, was found to upregulate TNIP1 expression in agreement with our previous findings of ATRA regulation of the TNIP1 promoter [206]. Together, this led Erdei and colleagues [205] to suggest a role for TNIP1 as an active repressor in the anti-inflammatory mechanism of ATRA. Broad transcriptional responses induced by ATRA beyond TNIP1 may limit it as a ready pharmacologic avenue to anti-inflammatory therapy. To that end however, it does support investigations of more TNIP1-specific routes to inducing its expression or reducing its inflammation-associated degradation in keratinocytes and other cell populations.

**Table 3:** Summary of epidermal or keratinocyte presentation from example alterations in genes associated with limiting or repressing cytoplasmic inflammatory signaling.

Gene ^#^	Alteration *	Keratinocyte/Epidermal or Other Presentation ^	Refs.^$^
*OTULIN*	HG: mutations	ORAS/AIPDS; IMD107; systemic inflammation, panniculitis, and rash	[91,92,93]
*Otulin*	mEKO: K14-Cre-mediated deletion	Early postnatal focal cutaneous inflammation, epidermal hyperplasia, reduced differentiation, and wound healing defects; increased cutaneous immune-cell recruitment	[100,101]
*OTULIN*	cKCKD	OTULIN-deficient HaCaTs; hypersensitivity to TNFα-induced necroptosis	[99]
*CYLD*	HG: mutations	Skin appendage neoplasms	[106,107]
*CYLD*	cKCOV: WT protein	WT overexpression in HaCaTs; increased levels of early differentiation markers	[114]
*CYLD*	cKCOV: C601S mutant	Deubiquitinase mutant expression in HaCaT keratinocytes; disorganized in vitro stratification, increased NF-kB signaling, and TNFα production	[109,114]
*CYLD*	mETG: WT protein	Level of late differentiation increased and reduction in NF-κB signaling	[109,116]
*CYLD*	mETG: C601S mutant	Increased phospho-p65, TNFα, and IL-6 with slower phospho-p65 resolution	[109,116]
*Cyld*	mEKO: K14-Cre-mediated deletion	Heightened levels of K63 polyubiquitinated TRAF6 and phospho-IκBα, cylindroma and trichoepithelioma development with DMBA and TPA challenge	[112]
*TNFAIP3* ^+^	HG: SNPs	Numerous inflammatory diseases (see text)	[120,121,122]
*TNFAIP3* ^+^	HG: mutations	A20 (see text); skin rash and inflammation in several other organs and tissues	[123]
*TNFAIP3* ^+^	cKCOV	A20 overexpression in NHEKs; inflammatory genes repressed upon IL-17A or TNFα challenge	[140]
*TNFAIP3* ^+^	cKCKD	A20-deficient human gingival keratinocytes; increased apoptosis when challenged with both TNFα and cycloheximide	[155]
*Tnfaip3* ^+^	mEKO: K14-Cre-mediated deletion	Keratinocyte hyperproliferation	[141]
*Tnfaip3* ^+^	mEKO: K14-Cre-mediated deletion	Enhanced inflammatory gene expression in cultured keratinocytes under basal conditions and IL-17A stimulation	[138]
*TNIP1*	HG: SNPs	Numerous inflammatory diseases (see text)	[179,180,181,182]
*TNIP1*	HG: mutations	SLE risk haplotype (P151A), unknown functional consequence	[182]
*TNIP1*	cKCOV	TNIP1 overexpression in NHEKs; repression of inflammatory genes upon IL-17A and TNFα challenge	[140]
*TNIP1*	cKCKD	TNIP1-deficient HaCaT keratinocytes; increasedexpression of inflammatory genes upon poly(I:C) exposure	[68,165]
*Tnip1*	mKO and mEKO: K14-Cre-mediated deletion	Psoriasis-like skin symptoms upon imiquimod exposure	[175]

^#^ Mouse genes (all italics, first letter capitalized); human genes (all italics, all capitals). * Alteration. HG: human genome variation; mKO, mouse germline null knockout; mEKO, mouse epidermal keratinocyte-selective knockout; mETG, epidermal keratinocyte-directed transgene overexpression; cKCKD; cultured keratinocyte knockdown; cKCOV, cultured keratinocyte overexpression. Descriptions of spontaneous human or murine genes are representative and not necessarily inclusive of all reports. ^ Highlights of cutaneous or cell phenotype, as in text, when available from the literature. ^+^ A20-encoding gene in humans (*TNFAIP3*) and mice (*Tnfaip3*). ^$^ For initial report or other description of alteration.

## 4. Conclusions/Perspectives

Skin infection and tissue damage can individually and together be the responsible events for protein and nucleic acid debris which trigger keratinocyte and immune-cell production of diverse cytokines. These in turn serve as ligands for cell-membrane receptors capable of triggering inflammatory signals amplifying and cascading through the cytoplasm. To limit initial signaling and facilitate return to tissue homeostasis, there are several proteins along the cytoplasmic inflammation signaling pathway that appear poised to quell the pro-inflammatory activity of select transmembrane receptors, their downstream kinases, and inflammasome proteins. Functioning within this cytokine- and PAMP/DAMP-induced pathway, the proteins SHARPIN, HOIL-1, HOIP, OTULIN, CYLD, A20, and TNIP1 are unified in their repression of signaling through sometimes overlapping, but also independent, mechanisms, as we presented above. Their shared characteristic of interaction with polyubiquitin chains on other proteins to bring about this repression also reflects the importance of multiprotein assemblages in inflammation repression as well as signal progression in the cytoplasm.

The proteins highlighted here limit or repress inflammation and are a naturally occurring counterbalance to proteins amplifying or driving its progression. While genomic alterations in, or absence of some, inflammation-repressing proteins such as A20 can lead to monogenic inflammatory diseases, the deficiency or defective function of others described here may add to increased susceptibility, prolonged duration, or reduced resolution of acute and chronic cutaneous inflammatory diseases. Though inhibition of receptors and enzymes activating inflammatory signaling has been a classical approach to limiting inflammation, proteins otherwise involved in regulating signaling may represent an important emerging complement for pharmacological investigations that could lead to clinical control of inflammation. For instance, LUBAC inhibitors targeting HOIP [207,208] have been isolated, with at least one inhibiting HOIP at a submicromolar range and reducing inflammation in a murine psoriatic model [209]. Those proteins that are ubiquitin-readers or scaffolds for assembly of signaling complexes may be more difficult to target compared to the catalytic control of others. A small-molecule chaperone approach may be a productive avenue. Separately, a distinct tactic could be increasing the expression of inflammation repressors. The plant-derived compound formononetin increases A20 gene expression and subsequent protein levels with a reduction in inflammatory markers in both keratinocyte culture and mouse models of atopic dermatitis [210]. The findings for HOIP and A20 illustrate that opportunities for experimental investigation of, and clinical control over, cutaneous inflammation exist well outside the current armamentarium of cytokine- and receptor-targeting monoclonal antibodies and enzyme inhibitors.

## Figures and Tables

**Table 1:** Pathophysiology of select inflammatory diseases from the text.

Disease	Pathophysiology
Psoriasis (plaque)	Raised, scaly, erythematous regions; over-production of and hyperresponsiveness to cytokines (e.g., TNFα, IL-23, and IL-17); increased numbers of immune cells; keratinocyte hyperproliferation and altered differentiation
Atopic dermatitis	Epidermal barrier defect, increased permeability of irritants; Th1 overactivity causes chronic lesions and pruritus; scratching skin stimulates keratinocyte release of inflammatory cytokines (e.g., TNFα and IL-1, IL-6)
Systemic lupus erythematosus	Cutaneous features: immune-cell infiltration; hyperkeratosis; autoinflammation and vasculopathy presenting as a malar (facial) rash, frequent photosensitivity; keratinocyte DAMP release and interferon production
Systemic sclerosis	Vascular insult progresses to chronic tissue hypoxia concurrent with increased pro-fibrotic cytokines (e.g., TGF-β, IL-5, and IL-13) and decreased anti-inflammatory cytokine (e.g., IFN-γ) production; autoimmunity
Cylindromatosis	Skin-appendage-derived tumors including cylindromas, trichoepitheliomas, and spiradenomas; usually benign but can progress to malignancy
Haploinsufficiency A20	Clinically variable presentation similar to Behcet’s disease; hyperkeratosis; pustular rash, acne, dermal abscesses, and oral and genital ulcers; hyperresponsive to stimuli for production of cytokines, e.g., IL-6

## Data Availability

Data sharing not applicable. No new data were created or analyzed in this study. Data sharing is not applicable to this article.
